# In Situ Growth of CuWO_4_ Nanospheres over Graphene Oxide for Photoelectrochemical (PEC) Immunosensing of Clinical Biomarker

**DOI:** 10.3390/s20010148

**Published:** 2019-12-25

**Authors:** Zaheer Abbas, Razium Ali Soomro, Nazar Hussain Kalwar, Mawada Tunesi, Magnus Willander, Selcan Karakuş, Ayben Kilislioğlu

**Affiliations:** 1 Beijing Advanced Innovation Centre for Soft Matter Science and Engineering, Beijing University of Chemical Technology, Beijing 100029, China; zaheer0714@mail.buct.edu.cn; 2 State Key Laboratory of Organic-Inorganic Composites, Beijing Key Laboratory of Electrochemical Process and Technology for Materials, Beijing University of Chemical Technology, Beijing 100029, China; mawadatunsi@gmail.com; 3 Institute of Chemistry, Shah Abdul Latif University Khairpur, Khairpur 66020, Pakistan; nazarkalwar@gmail.com; 4 Department of Science and Technology, Campus Norrkoping, Linkoping University, SE-60174 Norrkoping, Sweden; 5 Department of Chemistry, Faculty of Engineering, Istanbul University-Cerrahpaşa, Avcılar, Istanbul 34320, Turkey; selcan@istanbul.edu.tr (S.K.); ayben@istanbul.edu.tr (A.K.)

**Keywords:** CuWO_4_ nanostructures, procalcitonin, graphene oxide, immunosensor

## Abstract

Procalcitonin (PCT) protein has recently been identified as a clinical marker for bacterial infections based on its better sepsis sensitivity. Thus, an increased level of PCT could be linked with disease diagnosis and therapeutics. In this study, we describe the construction of the photoelectrochemical (PEC) PCT immunosensing platform based on it situ grown photo-active CuWO_4_ nanospheres over reduced graphene oxide layers (CuWO_4_@rGO). The in situ growth strategy enabled the formation of small nanospheres (diameter of 200 nm), primarily composed of tiny self-assembled CuWO_4_ nanoparticles (2–5 nm). The synergic coupling of CuWO_4_ with rGO layers constructed an excellent photo-active heterojunction for photoelectrochemical (PEC) sensing. The platform was then considered for electrocatalytic (EC) mechanism-based detection of PCT, where inhibition of the photocatalytic oxidation signal of ascorbic acid (AA), subsequent to the antibody–antigen interaction, was recorded as the primary signal response. This inhibition detection approach enabled sensitive detection of PCT in a concentration range of 10 pg·mL^−1^ to 50 ng.mL^−1^ with signal sensitivity achievable up to 0.15 pg·mL^−1^. The proposed PEC hybrid (CuWO_4_@rGO) could further be engineered to detect other clinically important species.

## 1. Introduction

The recent up-gradation in photoelectrochemical (PEC) sensors based on their synergic combination with advanced nanomaterial has proven effective in achieving robust signal sensitivities for low-concentration clinical species. In particular, the detection of cancer biomarkers, whose precise detection at a low concentration is crucial for timely diagnosis and therapeutics [1,2]. Despite the rapid progress in PEC engineering, the fast charge-carrier recombination and limited immobilization area, particularly in the case of bio immunosensors, are major challenges that restrict the construction of an efficient PEC biosensor [3]. In this context, various combinations of materials, including metal–organic frameworks (MOFs), semiconductor/carbon composites, transition metal dichalcogenides, and their hybrids, have been proposed to advance the PEC sensors performance [4,5,6]. Here, the use of hierarchical structures, complex morphologies of nanomaterials have shown relatively superior performance compared with their traditional counterparts. Particular, in terms of electro-catalytic activity, the complex morphologies are known to exhibit superior performances owing to the mishmash of free surface energy, the charge density concentrated at the corners of the complex morphology, and the larger total surface area [7,8,9,10,11]. On the other hand, the use of heterojunction is also a promising approach to acquire a high throughput signal. However, The improved efficiencies of these heterojunctions, are often associated with procedural complexity, high fabrication cost, and biocompatibility issues, which restrict their practical use in developing a single PEC platform capable of detecting clinically-important molecules [12,13,14].Here, the combination of highly photo-active nanostructures with a conductive substrate such as graphene, to support fast-charge transportation and greater immobilization of bio-reagents could be a viable option [15,16,17,18]. Among the many metal oxides and tungstates, copper tungstate (CuWO_4_) based on its suitable band-gap, high redox activity, and excellent catalytic properties is widely known for its use in photo-catalysis, water-splitting reaction, and fuel cell reactions. However, the use of CuWO_4_ as a photo-active transducer in PEC immunosensing still remains at an early stage [19]. Although CuWO_4_, compared with other tungsten materials, is more resilient to the photo-corrosion, and with its inherent capability to harvest visible light and chemical stability, it has the potential to be utilized as a photo-electrode. However, the relatively slow charge-carrier separation/mobility and their recombination limit the photocatalytic efficiency of CuWO_4_ [20]. In addition to high photo-activity, designing a nanoscale morphology that could support greater immobilization and maintain a good redox activity to generate a boosted signal response is yet another challenge when designing efficient PEC biosensor [21]. In regard to clinical biomolecules, procalcitonin (PCT) is an essential clinical biomarker primarily used for the diagnosis of bacterial infections. Rapid and selective detection of PCT from human serum samples would be beneficial for an early diagnosis, thereby decreasing the overall response time for the treatment. Contrary to the conventional approaches such as radioimmunoassay [4], colorimetric immunoassay [5], and enzyme-linked immunosorbent assay [6], the electrochemical approach designed for the detection of such molecules offers the advantage of high sensitivity, rapid detection time, and the capability to be transformed into a miniaturized device for field portability [7,8]. As the concentration of PCT in normal blood is less than 0.1 ng mL^−1^ [3], a highly sensitive electrochemical system is required for its accurate detection.

In this work, we describe the construction of an efficient PEC immunosensing platform for sensitive detection of the PCT biomarker. The platform is based on the hybrids of photo-active CuWO_4_ nanosphere and reduced graphene oxide layers (rGO). Here, CuWO_4_ nanospheres were directly grown (in situ) over rGO layers, constructing a highly efficient photo-active heterojunction with minimum charge-carrier recombination and high photocatalytic activity. Taking advantage of this synergic combination, an electrocatalytic (EC)-mechanism based PEC sensor was constructed, where inhibition in the measured photoresponse of ascorbic acid (AA), subsequent to the protein–antigen interaction, was found to be linear against the added concentration of the PCT biomarker. This detection strategy produced a robust signal response for PCT in the concentration range of 10 pg.mL^−1^ to 50 ng.mL^−1^, with signal sensitivity achievable up to 0.15 pg·mL^−1^. The described strategy in combination with the proposed electrode design could be configured with a variety of functionalities and receptor moieties for selective and sensitive detection of other clinically significant biomolecules.

## 2. Materials and Methods

The chemical and reagents were used as supplied. Sodium tungstate dihydrate, copper chloride, ethanol, and sodium citrate were obtained from Sigma-Aldrich (Darmstadt, Germany). The PCT antibodies, antigen, and bovine serum albumin (BSA) were supplied from Ye Xiang Bio. Co. Ltd. (Hangzhou, China). Sodium hydroxide (NaOH), phosphate-buffered saline (PBS; pH 7.0), and HCl (0.1 M) were from Thermo Fisher Scientific. Ascorbic acid and cocamidopropyl betaine (CAPB) was purchased from Sigma Aldrich (Darmstadt, Germany). The electrochemical experiments were performed using the CHI760D electrochemical workstation of Chenhua Co., Ltd. (Shanghai, China), equipped with a PLS-SXE 300 xenon lamp serving as a light source for PEC analysis. To avoid cell heating, the distance between the lamp and photo-cell was maintained at 12 cm. The morphological evaluation was obtained using field emission scanning electron microscopy (FE SEM) analysis (Oberkochen, Zeiss Sigma). X-ray powder diffraction (XRD) was performed with a D8 Advance X-ray diffractometer (Bruker AXS, Bremen, Germany). X-ray photoelectron spectroscopy (XPS; Scienta ESCA200) and raman spectroscopy (WITech alpha300) were carried for the phase confirmation analysis. In this study, a modified glassy carbon electrode (GCE), a saturated calomel electrode (SCE), and a platinum wire electrode (PE) were used as working, reference, and counter electrodes, respectively.

### 2.1. In Situ Growth of CuWO_4_ Nanospheres over Graphene Oxide (rGO)

Initially, reduced graphene oxide (rGO) flakes were synthesized using a well-known modified Hummers method [15]. The obtained rGO flakes were then dispersed (0.1 mg) in an aqueous solution of 0.1 M CuCl_2_.2H_2_O (50 mL) previously containing (0.1 M) of CAPB, used as a template. The mixture was then gradually introduced with 0.1 M of Na_2_WO_4_.2H_2_O under constant stirring condition. The suspension was later transferred to a Teflon lined autoclave and subjected to heat treatment at 90° for 12 h, to facilitate the growth of CuWO_4_ nuclei. The final precipitates were thoroughly washed and dried at 60° before utilizing them for the construction of PEC platform. The formed hybrid nanostructures are designated as CuWO_4_@rGO throughout the manuscript.

### 2.2. Developing a PEC Immunosensing Platform

**Figure 1:**
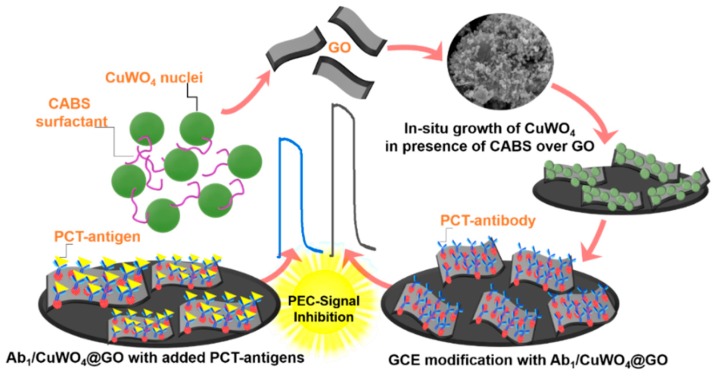
A generalized scheme, portraying in situ growth of CuWO_4_ nanospheres over reduced graphene oxide (rGO) flakes and the hybrids use in photoelectrochemical (PEC) electro-catalytic immunosensing of procalcitonin (PCT) biomarker where signal inhibition is the consequence of the antibody–antigen reaction occurring at the surface of CuWO_4_@rGO hybrids. CAPB, cocamidopropyl betaine, GCE, glassy carbon electrode.

Figure 1 shows the stepwise process adopted for developing an immunosensing platform using CuWO_4_@rGO hybrids. The surface of the GCE was initially polished with alumina powders (0.03 and 0.05 μm, respectively) and then washed with de-ionized water. First, a transducing platform was constructed by depositing dispersion (0.1 mg/5 mL methanol) of CuWO_4_@rGO hybrid over the pre-polished GCE surface. This layer was then allowed to dry under the nitrogenous atmosphere. To integrate the bio-recognition element, the electrode was then incubated with 5 μL of optimum 1.0 μ gmL^−1^ capture antibodies of PCT (Ab_1_-PCT) and then by 2 μL of 1% BSA to block the available active sites, followed by gently rinse before usage (Figure S1a). This electrode was designated as Ab_1_/CuWO_4_@rGO-GCE and stored at 4 °C before using it for immunosensing. To detect the PCT antigen, the Ab_1_/CuWO_4_@rGO-GCE was incubated with different concentration of PCT in the range of 10 pg·mL^−1^ to 50 ng·mL^−1^ separately.

### 2.3. PCT Detection from Simulated Blood Plasma

To ensure the analytical workability of the designed PEC sensors, the detection of PCT was also performed in simulated blood plasma (SBP). To simulate the real plasma-like characteristics, abundant proteins such as 40 mg of albumin, 10 mg of immunoglobulins, and 2.5 gm of fibrinogen were diluted in 10 mL of PBS solution (pH 7.0) to mimic their average plasma concentration. The SBP was then used in the quantification of different PCT concentrations in the range of 5 to 25 ng·mL^−1^ using designed PEC biosensors. To further validate the performance, the spiked samples were also quantified using the standard ELISA technique.

## 3. Results and Discussions

### 3.1. Characterization of CuWO_4_@rGO Hybrid

**Figure 2:**
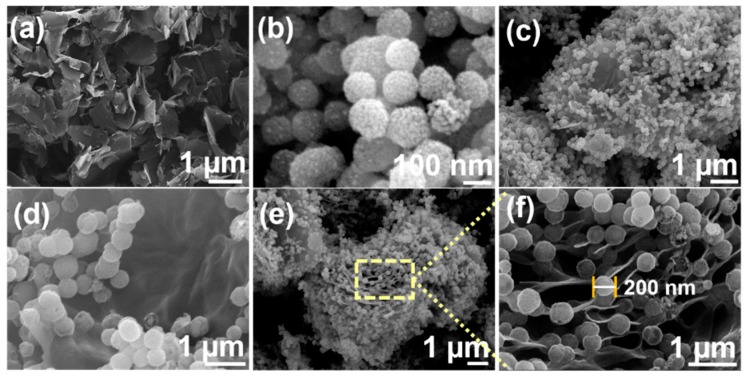
Scanning electron microscopy (SEM) images for (**a**) rGO flakes; (**b**) pristine CuWO_4_ nanospheres; (**c**–**e**) CuWO_4_@rGO hybrids; and (**f**) high-resolution capture of the section showing interface formation between nanospheres and rGO edges.

**Figure 3:**
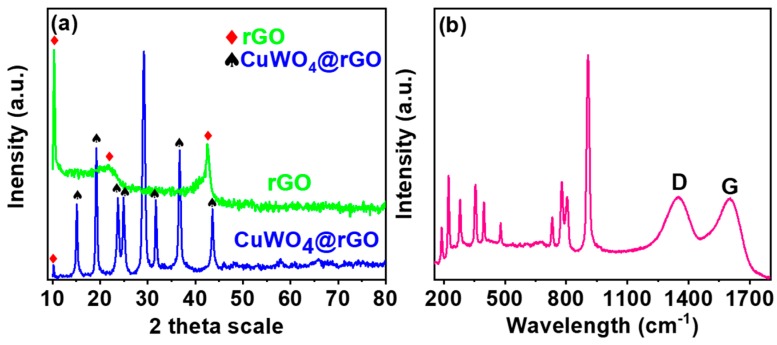
(**a**) X-ray powder diffraction (XRD) pattern recorded for CuWO_4_@rGO hybrids in reference to pristine rGO; (**b**) the Raman spectra of the CuWO_4_@rGO hybrids with prominent D and G bands typical of rGO.

Figure 2a–f shows the SEM micrographs obtained for pristine rGO, CuWO_4_, and the in situ grown CuWO_4_@rGO hybrids. The rGO flakes with a crumpled layer-like morphology could be seen in Figure 2a. Contrary to rGO, the pristine CuWO_4_ possessed a distinctive spherical shape. The CuWO_4_ spheres were primarily composed of tiny nano-boulders, which in presence of CAPB, self-assembled into 3D nanospheres (Figure 2d–f). The average size of the spheres was within the range of 190 to 220 nm, where the average size of the boulders was determined to be in the range of 10 to 25 nm. The formed morphology could be attributed to the growth controlling and directing capabilities of cocamidopropylbetaine (CAPB) surfactant. The in situ growth of CuWO_4_ nanospheres over rGO flakes could be observed in Figure 2c–f. A significant portion of the flakes could be seen occupied by CuWO_4_ nanospheres, where a high-resolution scan, shown in Figure 2f, confirms the growth of nanospheres along the edges of the flakes. Unlike conventional hybrids, where aggregation usually results in a complete mishmash of the particles. The nanospheres, in this case, have maintained their structural integrity without any structural collapse. Such structural features are highly beneficial in achieving enhanced electro-catalytic redox characteristics based on their exposed active sites and higher surface area. In addition, the nanospheres could be seen to be connected at the edge of rGO flakes, which is beneficial in achieving faster charge-transportation at the electrode interface. Figure 3a shows the XRD pattern of CuWO_4_@rGO hybrids, in reference to its compositional counterparts.

**Figure 4:**
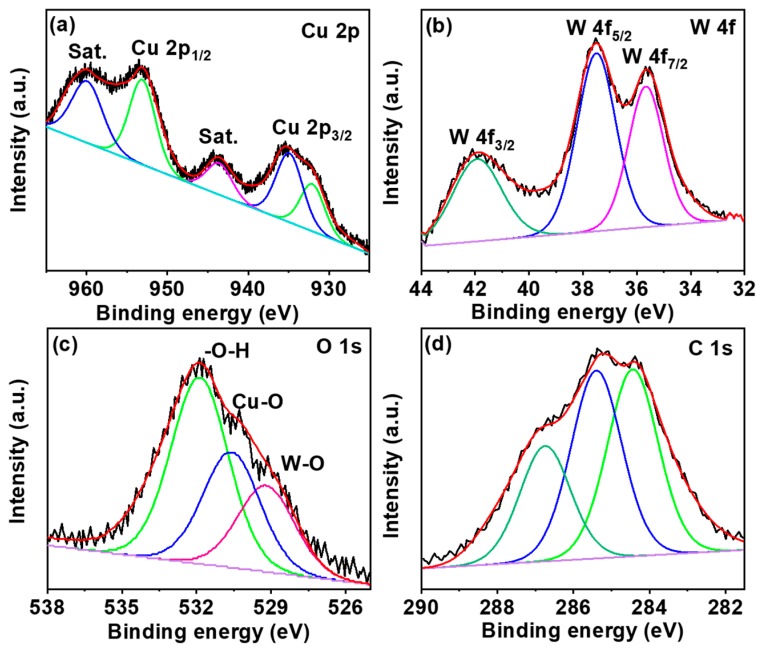
X-ray photoelectron spectroscopy (XPS) analysis of CuWO_4_@rGO hybrids (**a**) high-resolution Cu 2P; (**b**) W4f; (**c**) O1s; and (**d**) C1s elementals fittings.

The XRD pattern for rGO, CuWO_4_, and its hybrid is shown in Figure 3a. In resemblance to the rGO with a representative 10.3°, the XRD pattern for CuWO_4_@rGO hybrids also consist of this peak with a slightly declined intensity. The characteristic peaks for CuWO_4_ were observed at 15.1°, 19.2°, 23.8°, 24.9°, 29.2°, 31.6°, 36.7°, and 43.8°, indexed to the (010), (001), (110), (0 11), (111), (111), (200), and (121) planes of CuWO_4_ triclinic phase, respectively, as standardized against (JCPDS No 80-1918) [22]. The formation of the hybrid was further confirmed from Raman analysis. The corresponding spectra (Figure 3b) consists of the typical CuWO_4_ peaks along with prominent D and G bands at 1350 and 1605 cm^−1^, respectively [23]. The sharp peak around 900 cm^−1^ is attributed to the W–O vibration from the tungstate of CuWO_4_. The compositional purity of the hybrid was assessed from XPS analysis. The high-resolution de-convoluted profiles for the major components are shown in Figure 4a–d. The Cu 2p spectrum consists of two major peaks at 932.8 and 952.9 eV attributed to the core Cu^2+^ of the CuWO_4_@rGO hybrid. The presence of the satellite peak confirms the presence of copper as in Cu (II) form, whereas the intense binding energy of Cu 2p_3/2_ suggests that the Cu^2+^ ions are surrounded by W–O from WO_4_^−2^ ions. The W4f spectrum exhibits two major peaks at 37.1 eV and 34.8 eV, indicating the presence of tungsten as in the W^6+^ state. The O1s spectrum is resolved into three peaks (531.6, 530.1, and 529.3 eV) related to surface adsorbed hydroxide, Cu–O, and W–O bonds. The C1s spectrum confirms the presence of carbon (284.2 eV), epoxy carbon (285.2 eV), and carboxyl (286.1 eV) species from the rGO counterpart of the CuWO_4_@rGO hybrid [24]. The XPS analysis in support of XRD and Raman confirms the successful formation and compositional purity of CuWO_4_@rGO hybrids.

### 3.2. Photoelectrochemical Performance of CuWO_4_@rGO Hybrids

**Figure 5:**
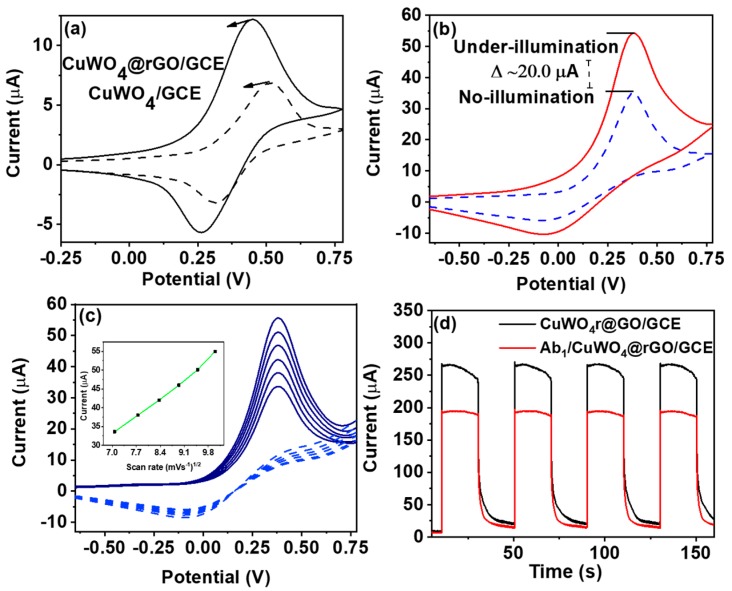
(**a**) CV behaviour of the CuWO_4_@rGO in reference to pristine CuWO_4_; (**b**) electro-catalytic oxidation of ascorbic acid (AA) (0.1 mM) using CuWO_4_@rGO/GCE both under illumination and no illumination conditions; (**c**) peak current variation against scan rate in range of 50 to 100 mVs^−1^ with inset depicting linearity between current response and square root of scan rate; (**d**) photo-response of CuWO_4_@rGO/GCE and Ab_1_/CuWO_4_@rGO/GCE with 30 s cut-off cycle for 150 s.

To evaluate the electro-catalytic performance of CuWO_4_@rGO hybrids, CV measurements were recorded using 0.1 mM of ascorbic acid (AA). Figure 5a shows the measured CV curves, where the CuWO_4_@rGO hybrid exhibits a relatively higher current response at lower potential value compared with its pristine counterparts. The obtained response of CuWO_4_@rGO hybrid against AA was further recorded with different scan rates in the range of 5 to 50 mVs^−1^. As seen (Figure 5c), the anodic peak could still maintain peak-shape characteristics at higher scan rates, reflecting the electrochemically reversible charge-transfer process of the diffused redox species, that is, AA. The observed linearity between measured current response and the square root of the scan rate (inset of Figure 5c) further defines this interfacial redox process to be diffusion-limited. To ensure maximum signal throughput, the pH of AA was optimized. Figure S1b indicates that the maximum current output was achieved when AA was maintained at pH 7. The photocatalytic activity of CuWO_4_@rGO hybrid was also evaluated under the illumination condition (Figure 5b). As evident, CuWO_4_@rGO hybrid exhibits significantly high photocurrent response (∆I = 20 µA) under light. This increased current response could be explained using the EC-mechanism. In this case, the scavenger (AA), could readily oxidize over the surface of the CuWO_4_@rGO hybrid under the illumination condition, consuming the photo-generated holes, while at the same time promoting the electron transfer to the conduction band of CuWO_4_, and subsequently to the rGO layer. Here, rGO serves as a charge collector, facilitating the interfacial charge-transfer process.

**Figure 6:**
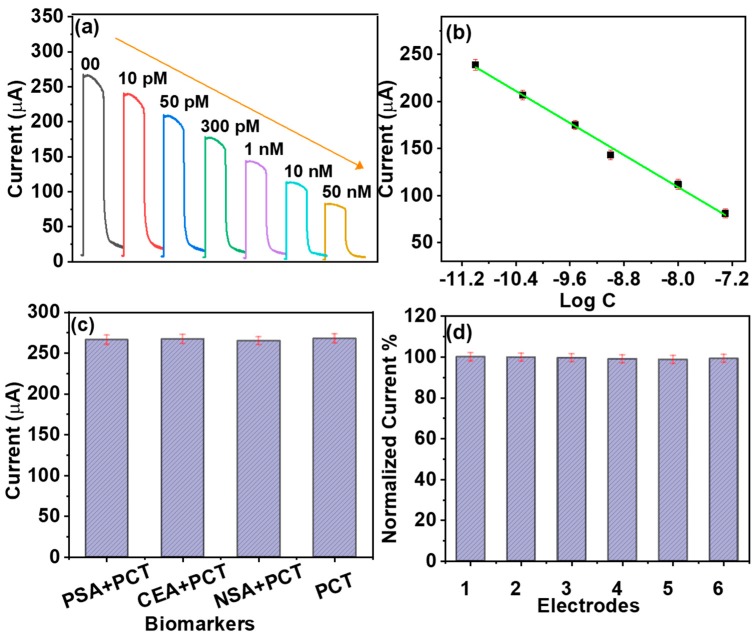
(**a**) Photo-current responses of Ab_1_/CuWO_4_@rGO/GCE against the different concentration of PCT in the range of 50 ng·mL^−1^ to 10 pg·mL^−1^; (**b**) the corresponding inhibition calibration plot; (**c**) the immunoreactivity of CuWO_4_@rGO towards PCT in the presence of different biomarkers; (**d**) the measured signal reproducibility for six similarly fabricated CuWO_4_@rGO electrodes against 10 ng·mL^−1^ of PCT, with 0.1 mM AA, in 0.1 M phosphate-buffered saline (PBS) electrolyte. PSA, prostate-specific antigen; CEA, carcinoembryonic antigen; NSA, neuron-specific enolase (NSA).

The increased photocurrent, in this case, confirms the oxidation of AA as a favourable process for the designed electrode, and thus AA could be anticipated as a potential redox mediator in the PEC immunosensor designed for the PCT biomarker. The current versus time measurements were carried to confirm the immobilization of biorecognition elements (PCT antibodies (Ab_1_)) over CuWO_4_@rGO/GCE (Figure 5d). The measurements were recorded in 0.1 mM AA with 0.1 M PBS (pH 7) with 30 s light on/off cycles for about 150 s. Here, Ab_1_/CuWO_4_@rGO/GCE exhibited a relatively lower current response compared with its protein-free counter-part. This decline is the consequence of active site blockage subsequent to the immobilization of insulating species (Ab_1_). Figure 6a shows the variation of photocurrent response with different concentrations of PCT standards. The measurements were carried under constant illumination, with a fixed potential of 0.15 V, 0.1 mM of AA as a mediator, and 0.1 M PBS (pH 7.0) as an electrolyte. This decline of the photoresponse with increasing concentration of PCT is the result of the immuno-complex formed between the PCT antibody–antigen at the CuWO_4_@rGO interface. In this case, the difference of photocurrent recorded before and after incubating the electrode with a specific concentration of PCT was taken as the primary signal. Figure 6b shows the corresponding calibration for the Ab_1_/CuWO_4_@rGO/GCE against the log concentration of PCT in the concentration range of 50 ng·mL^−1^ to 10 pg·mL^−1^. The detection limit of the devised PEC sensor was estimated to be 0.15 pg·mL^−1^ of PCT (3x S/N). The selectivity of the designed immunosensing platform was evaluated against different biomarkers including prostate-specific antigen (PSA), carcinoembryonic antigen (CEA), and neuron-specific enolase (NSA). The measurements were carried using the same conditions as mentioned in Section 2.2. The negligible current variation in the presence of interferents supports the robust immunoreactivity of the devised sensor towards the PCT antigen. The photo-response of six independently fabricated electrodes was also measured to evaluate signal reliability of the sensor. Figure 6d shows the normalized response Ab_1_/CuWO_4_@rGO/GCE against standard 10 ng·mL^−1^ of PCT, with 0.1 mM AA, in a 0.1 M PBS electrolyte. As expected, a negligible current variation was observed, which anticipates high signal reliability each time a new CuWO_4_@rGO/GCE is fabricated. The shelf-life of the devised sensor was evaluated for a period of seven days with measurements recorded at an interval of one day (Figure S1c). As evident, the photocurrent response of Ab_1_/CuWO_4_@rGO/GCE during the storage period does not change significantly, justifying the sensor’s capability to endure a longer service life. The practical workability of the designed PEC biosensor was evaluated by sensing PEC biomarkers from simulated blood plasma. The simulated blood plasma (SBP) comprised of human albumin solution, immunoglobulins, and fibrinogen maintained at their typical human blood plasma levels. The SBP was spiked with 5, 15, 20, and 25 ng·mL^−1^ of PCT and applied to ab_1_/CuWO_4_@rGO/GCE for detection. Figure S1d compares the obtained values with those determined using the ELISA technique. The close proximity between the measured values evidently supports the capability of the designed sensor to selectivity detect PCT without any effect from the complex biological medium, ensuring its workability in real-blood applications. Table 1 compares the analytical capability of the devised sensors with other electrochemical driven sensors previously reported for the quantification of PCT. As evident, the designed sensors offers competitive performance with additional merit of sophisticated integration within portable PEC devices, designed particularly for point-of-care clinical applications.

## 4. Conclusions

In this study, we describe a sensitive PEC platform devised for the detection of PCT clinical biomarkers. The platform comprises of highly photo-active CuWO_4_, grown in situ over conductive rGO layers. The CuWO_4_ with its template-based in situ growth approach adopts the morphology of 3D nanospheres, which are composed of tiny self-assembled nano-boulders with the size range of 2–5 nm. The in situ growth enables excellent interfacial contact between CuWO_4_ and rGO, allowing the construction of highly photoactive platform sensitive towards PCT biomolecules. The designed PEC sensor signal relies on the EC-mechanism, where signal inhibition observed for the photo-catalytic oxidation of AA, subsequent to the protein-antibody interaction, was taken as the primary PEC response. This strategy enabled sensitive inhibition signal linearity in the concentration range of 10 pg·mL^−1^ to 50 ng·mL^−1^ with a limit of detection of 0.15 pg.mL^−1^ for the PCT biomarker. Moreover, the devised platform, when tested in simulated blood plasma (SBP), demonstrated excellent reliability, anticipating its future potential in practical clinical applications.

## Figures and Tables

**Table 1:** Comparison of devised sensors with previously reported electrochemical sensors designed for the quantification of procalcitonin (PCT).

Materials	Limit of Detection (LOD) (pg·mL^−1^)	Linear Range	Reference
Au@Ag heterojunction with CeO_2_-CuO	0.17	0.5 pg·mL^−1^ to 50 ng·mL^−1^	[25]
SWCNHs–HPtNPs/PAMAM hybrids	1.74	10 pg·mL^−1^ to 20 ng·mL^−1^	[26]
SWCNHs/ HPtCs hybrid	0.43	1.00 pg·mL^−1^ to 20 ng·mL^−1^	[27]
layer coated GC/ MWCNTs/CS/GA hybrid	0.5	0.01 to 350 ng·mL^−1^	[5]
PTC-DEPA/KCC-1 NCs	0.017	0.05 pg mL^−1^ to 10 ng mL^−1^	[1]
CuWO_4_@rGO	0.15	10 pg·mL^−1^ to 50 ng·mL^−1^	This work

SWCNHs, single-walled carbon nanohorns; HPTCs, hollow Pt chains; PAMAM, Poly(amidoamine); GC, graphene; MWCNT, multi-walled carbon nanotubes; CS, chitosan; GA, glutaraldehyde; rGO, reduced graphene oxide; PTC-DEPA/KCC-1, perylene-3,4,9,10-tetracarboxylic acid-N,N-Diisopropylethylenediamine/mesoporous fibrous silica.

## Supplementary Materials

The following are available online at https://www.mdpi.com/1424-8220/20/1/148/s1, Figure S1: In situ growth of CuWO_4_ nanospheres over graphene oxide for photoelectrochemical (PEC) immunosensing of clinical biomarker.
