# Supplementary material for: In Situ Growth of CuWO4 Nanospheres over Graphene Oxide for Photoelectrochemical (PEC) Immunosensing of Clinical Biomarker
Source: Sensors (Basel). 2019 Dec 25;20(1):148. doi: 10.3390/s20010148 (PMC6983212; doi:10.3390/s20010148)
Supplement: Supplementary file 1 [file sensors-20-00148-s001.pdf]

Supplementary Data

In situ growth of $CuWO_4$ nanospheres over graphene oxide for photoelectrochemical (PEC) immunosensing of clinical biomarker

Figure S1

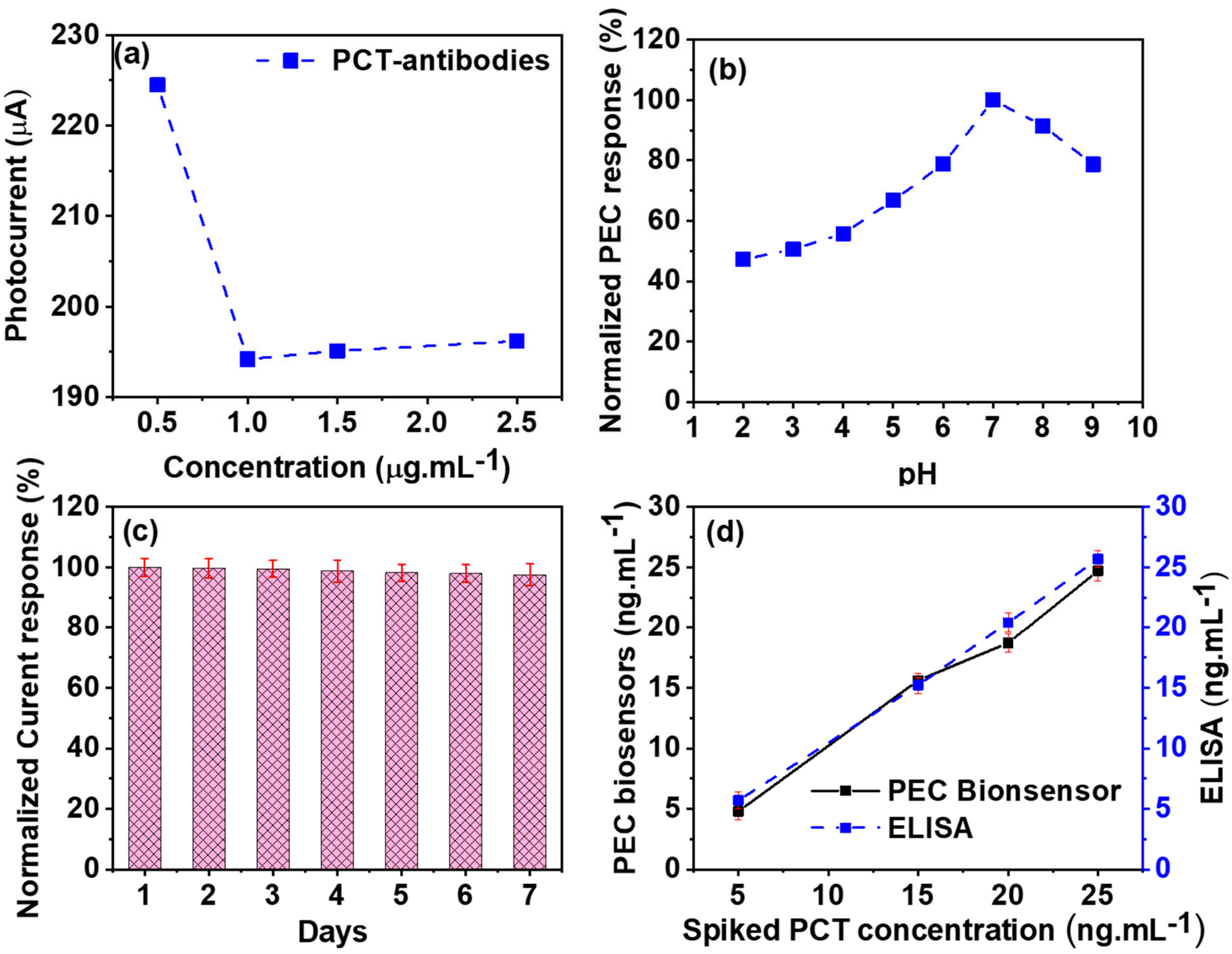

Figure S1. (**a**) photocurrent optimization of $Ab_1/CuWO_4@rGO/GCE$ against different concentration of PCT-antibodies, (**b**) variation of $CuWO_4@rGO/GCE$ photocurrent against different pH of AA, (**c**) shelf-life evaluation of devised electrode during 1 week of measurement, (**d**) the comparison of the PCT concentration measured using designed PEC sensor and standard ELISA within simulated blood plasma (SBP).
